# Increasing Viscosity Helps Explain Locomotor Control in Swimming *Polypterus senegalus*

**DOI:** 10.1093/iob/obab024

**Published:** 2021-08-10

**Authors:** K Lutek, E M Standen

**Affiliations:** Department of Biology, University of Ottawa, 30 Marie-Curie Private, Ottawa, ON K1N 6N5, Canada; Department of Biology, University of Ottawa, 30 Marie-Curie Private, Ottawa, ON K1N 6N5, Canada

## Abstract

Locomotion relies on the successful integration of sensory information to adjust brain commands and basic motor rhythms created by central pattern generators. It is not clearly understood how altering the sensory environment impacts control of locomotion. In an aquatic environment, mechanical sensory feedback to the animal can be readily altered by adjusting water viscosity. Computer modeling of fish swimming systems shows that, without sensory feedback, high viscosity systems dampen kinematic output despite similar motor control input. We recorded muscle activity and kinematics of six *Polypterus senegalus* in four different viscosities of water from 1 cP (normal water) to 40 cP. In high viscosity, *P. senegalus* exhibit increased body curvature, body wave speed, and body and pectoral fin frequency during swimming. These changes are the result of increased muscle activation intensity and maintain voluntary swimming speed. Unlike the sensory-deprived model, intact sensory feedback allows fish to adjust swimming motor control and kinematic output in high viscous water but maintain typical swimming coordination.

## Introduction

Coordinated locomotion in vertebrates is the result of central pattern generators (CPGs), top-down signals (i.e., from higher brain centers such as cerebral cortex and including integrated sensory information), and local sensory feedback. Sensory feedback relays information from the environment to the central nervous system, altering motor output and ultimately fine-tuning locomotor performance. Higher order senses like vision relay environmental information to CPGs via the brain (e.g., cortex/pallium) (Severi et al. 2014; Bollmann 2019). Local sensory feedback such as proprioception (e.g., muscle stretch receptors) and other more reflexive systems relay information directly to CPGs creating more immediate changes in motor output (Grillner et al. 1984; Viana di Prisco et al. 1990; Vinay et al. 1996). While each of these components is essential to adaptive, flexible locomotion (Goulding 2009; Garcia-Campmany et al. 2010; Kiehn 2016), how different sources of sensory feedback (either top-down or local) integrate and modulate locomotor behavior remains uncertain.

Fish use a variety of senses to tune their movements to their environment. Perturbations in flow demonstrate that fish use vision and lateral line sensing (both top-down sensory feedback systems) to control locomotion (Liao 2007). Although little is known about proprioception in fishes, new research suggests that fish do rely on proprioceptive feedback in both the spinal cord and fins to fine-tune motor control (Henderson et al. 2019; Aiello et al. 2020). Further, since putative vertebral stretch receptor cells have been found in lamprey as well as a variety of basal vertebrates including snakes, salamanders and elasmobranchs, it is likely that bony fishes also possess such cells (Massarelli et al. 2017). We can gain insight into how these sensory systems control locomotion by altering environmental conditions and watching how animal behavior changes. Highly viscous environments alter the mechanical forces experienced by an animal by increasing the boundary layer surrounding a swimming fish and decreasing the relative importance of inertial forces (lowering Reynolds number [Re]). In other words, high viscosity systems are dampened, which may impact sensory feedback. Further, increased efforts are likely required to initiate and maintain motion in these environments, necessitating changes in motor control and affecting swim performance (Horner and Jayne 2008; Tytell et al. 2010).

While most studies that have manipulated viscosity are aimed at questions outside of motor control (e.g., cold temperature metabolism, performance limits), they all indirectly suggest that sensory feedback is essential for adjusting to novel environmental mechanics (Fuiman and Batty 1997; Johnson et al. 1998; Hunt von Herbing and Keating 2003; McHenry and Lauder 2005; Horner and Jayne 2008; Danos 2012; Danos and Lauder 2012). Indeed, anguilliform swimming likely requires additional effort to maintain swim speed in a high viscosity environment, as is seen in lungfish swimming in high viscosity (Horner and Jayne 2008). In addition, computer simulations of anguilliform swimming that lack sensory feedback, but maintain internal mechanics and muscle activation frequency, display dampened kinematics and decreased swim speed when environmental viscosity is increased (Tytell et al. 2010). These simulations also develop an increased phase lag between muscle activation and body curvature in a high viscosity environment. In contrast, a variety of intact fishes maintain voluntary swim speed as viscosity increases (steady swimming: Fuiman and Batty 1997; Johnson et al. 1998; Hunt von Herbing and Keating 2003; Horner and Jayne, 2008; and unsteady swimming: McHenry and Lauder 2005; Danos 2012; Danos and Lauder 2012). To maintain speed in an increased viscous force environment, fish must actively change their muscle activation patterns, as seen in lungfish, which increase their muscle effort (rectified integrated area of the electromyography signal, RIA) and experience the predicted increase in phase lag between electromyography (EMG) onset and maximum body curvature when swimming through viscous media (Horner and Jayne 2008). The signaling of this change in motor control is most likely due to one or many of the abovementioned sensory feedback systems. While muscle activation changes kinematic output, the altered forces in the environment passively constrain kinematics, as is seen in the reduction of speed and amplitude of motion in sensory-deprived computer simulations (Tytell et al. 2010). In this study, we measure muscle activation patterns and resultant kinematic performance of the elongate bony fish, *Polypterus senegalus*, in a series of different viscous regimes. *P.senegalus* is the most basal actinopterygian. It has a predominantly aquatic life history, but also has the ability to locomote on land. Because of its phylogenetic position, and its ability to locomote amphibiously, *P. senegalus* has become an interesting evolutionary model and a considerable amount is known about how it changes motor control between its walking and swimming gaits. This study aims to understand *P. senegalus*’ capacity to adjust to more subtle changes in environment, thus building a larger dataset with which to understand how sensory feedback and environmental forces impact motor control. By leaving sensory systems intact and manipulating the environment, we discuss how sensory feedback systems, combined with passive mechanical constraint, may be involved in motor control in intact animals.

## Materials and methods

### Animals

*P. senegalus* were acquired from the pet trade (AQUAlity Tropical Fish Wholesale Inc., Mississauga, ON, Canada). Fish were kept in individual recirculating (10% water change each week) flow-through tanks on a 12/12 light cycle at 25–26°C. Six fish (total length: 136.33 ± 1.32 mm; mass: 14.55 ± 0.54 g) were used for kinematic and EMG experiments. All fish swam in 1 cP (normal water viscosity) and 40 cP water. Three of these fish also swam in 5 cP and 10 cP water. Fish numbers were chosen based on variation seen in previous experiments. All experiments were performed according to University of Ottawa Animal Care Protocol BL-2069.

### High-speed videography

Water viscosity was altered by the addition of methyl cellulose (400 cP; M0262, Sigma-Aldrich) and measured before each experiment using either a S1 or S2 Shell Cup^®^ (Norcross Corporation, Newton, MA). Note that at the concentration necessary to achieve a viscosity of 40 cP (<1% w/w), we would expect the solution to exhibit Newtonian behavior (Herráez-Domínguez et al. 2005). Fish swam in a standing water tank (5.4 cm × 80 cm) and were filmed from below by a Photron Fastcam Mini UX (Photron USA Inc., San Diego, CA) at 250 frames per second. The height of the water for all trials was 5 cm. All experiments were run with room temperature water (23–24°C). Fish were in each condition for no more than 10 min to minimize stress and were given free access to the surface to breathe using their lungs, as desired. The order that the fish were exposed to each viscosity was randomized to minimize any order effects of the different conditions. Videos were analyzed only if the fish completed three or more steady locomotor cycles (tail fin beats) in a row (Movies S1 and S2). A total of 5–10 cycles were analyzed for each fish in each condition. The nose and tip of the caudal fin was digitized using DLT Data Viewer 6 (Hedrick 2008). Videos were binarized and fish midlines (from nose to tip of caudal fin) were automatically tracked using custom Matlab code. Frame numbers and *x*–*y* coordinates of the pectoral fin lobes when the fin began adduction and abduction were identified in FIJI (Schindelin et al. 2012).

### Kinematics analysis

**Fig. 1:**
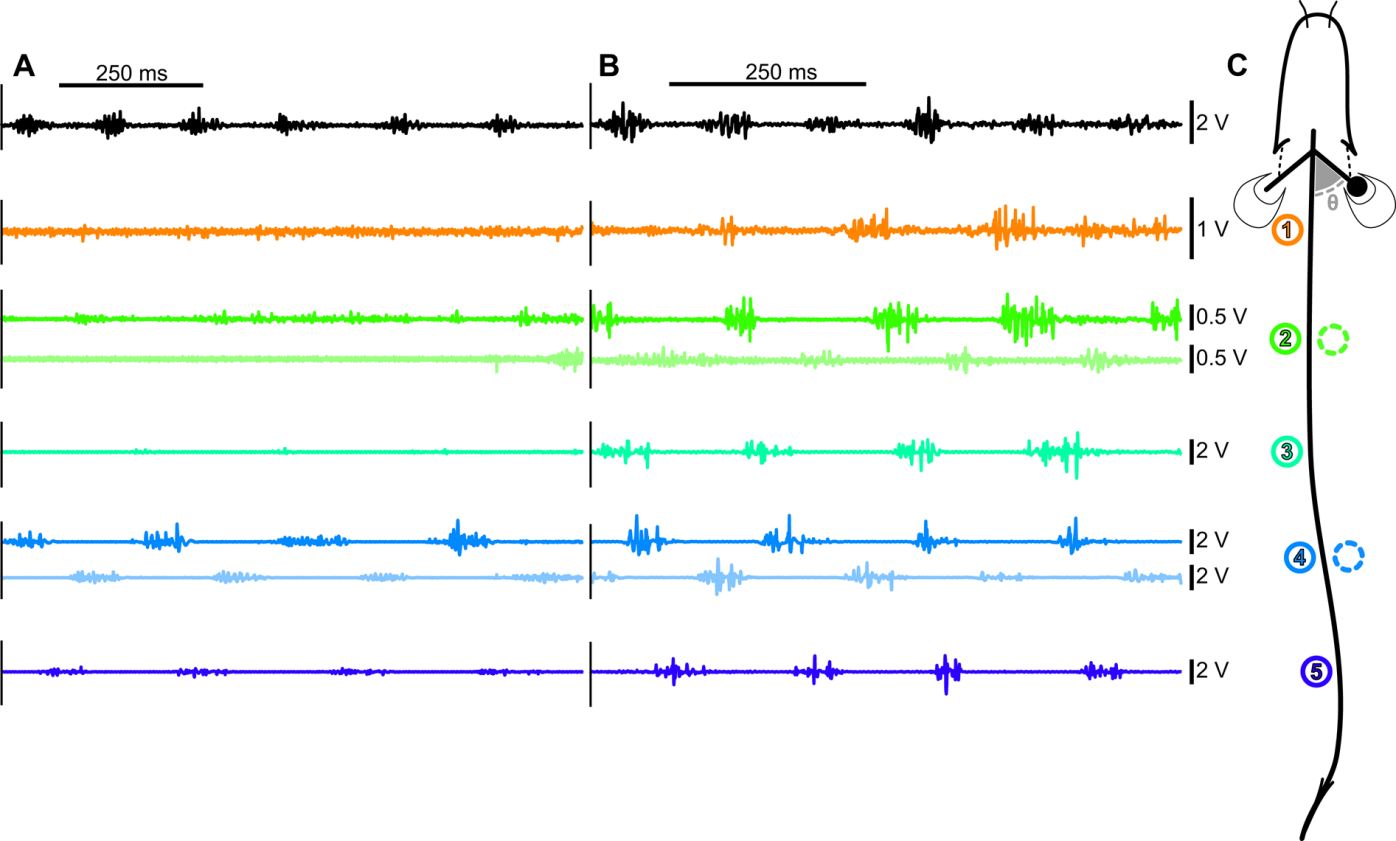
Representative traces of muscle activity, electrode position and pectoral fin angle. Muscle activity in 1 cP water (A) and 40 cP (B) for the pectoral fin and left-side body electrodes. Electrode position and pectoral fin angle calculation for all fish (C). Five electrodes were implanted on the left side of the body spaced equally between the posterior edge of the pectoral fin and the anterior edge of the anal fin. Two electrodes were implanted on the right side to match body positions 2 and 4 on the left side. One electrode was implanted in the adductor muscle of the right fin. Fin angle was calculated as the supplementary angle to that made by the head, 20% body length (BL; approximate position of the pectoral girdle) and the middle of the tip of the pectoral fin lobe (denoted θ in panel C). Pectoral fin adductor (black), electrode 1 on the left side (20.5 ± 0.008% BL; orange), electrode 2 on the left side (33.8 ± 0.015% BL; green), electrode 3 on the left side (47.6 ± 0.013% BL; cyan), electrode 4 on the left side (59.4 ± 0.012% BL; light blue), and electrode 5 on the left side (69.2 ± 0.013% BL; dark blue). Traces for electrodes on the right side are shown directly under the matching left-side trace at 50% transparency as indicated by the color matching positions in panel C.

The following variables were calculated for each video sequence: swim speed (BL s^–1^; BL, body length), maximum body curvature (calculated over 100 equal length segments along the entire length of the fish; BL^–1^), body wave speed (traveling wave of maximum body curvature passing along three sections of the fish: 35–55% BL, 55–75% BL, and 75–95% BL; BL s^–1^), body wave frequency (cycles s^–1^), fin frequency (defined by the start of pectoral fin adduction; cycles s^–1^), fin angle at start of adduction (supplementary angle to the angle between the tip of the nose, back of skull and tip of the pectoral fin lobe; radians; Fig. 1), fin angle at the start of abduction (radians; Fig. 1), and Re. Values for body curvature and body frequency were calculated at each position where they could be reliably detected. Therefore, body curvature was not calculated for the head or tail, and body frequency was not calculated for the head or the two most anterior electrode positions. All variables were calculated using custom code in Matlab (version R2018/2019, The MathWorks, Natick, MA).

### Electromyography

Prior to surgery, fish were lightly anesthetized in 200 mg L^–1^ buffered tricaine methanesulfonate (MS222, Syndel Laboratories Ltd., BC, Canada). During surgery, fish were kept moist and anesthetized with holding tank water and anesthetic. Two-pronged electrodes were fashioned out of 0.051 mm insulated bi-filament stainless steel wire (California Fine Wire Company, Grover Beach, CA, USA) and implanted in the fish with 27 gauge needles (Sigma-Aldrich). Electrodes were implanted in the red muscle zone <1 mm deep at five locations just above the lateral line down the left side of the body (just behind the pectoral fin base, 20.5 ± 0.008% BL; just anterior to the anal fin, 69.2 ± 0.013% BL; and three locations evenly spaced in between: 33.8 ± 0.015% BL, 47.6 ± 0.013% BL, and 59.4 ± 0.012% BL) (Fig. 1). To compare left- and right-side body muscle timing, two electrodes were placed on the body's right side at the second and fourth positions (34.6 ± 0.013% BL and 61.2 ± 0.012% BL, respectively) (Fig. 1). Finally, one electrode was placed in the right pectoral fin adductor muscle (Fig. 1). Electrode wires were secured to the dorsal finlets with suture to reduce strain and the fish was allowed to recover in tank water. EMG signals were recorded using a Grass P511 AC Amplifier (Grass Instrument Company, West Warwick, RI, USA), fed through a Powerlab 16/35 digital-to-analog converter (AD Instruments, Colorado Springs, CO, USA) and recorded in Lab Chart 8 (AD Instruments). Signals were recorded at 10 kHz with a 60 Hz notch filter to eliminate ambient electrical noise and a 40–4000 Hz band pass filter to eliminate movement artifacts, and analyzed with custom Matlab code. Video and EMG recordings were synchronized using an external trigger. Following euthanasia (MS222 417 mg L^–1^ in housing water), mass and length were recorded and electrode locations were confirmed by dissection.

To facilitate comparison of muscle intensity across individuals, we found the maximum value EMG recorded over all trials, for each electrode (EMG_ExpMax_). Rectified integrated area under the EMG curve (RIA) is reported as a percentage of theoretical maximum RIA for each muscle burst (theoretical maximum RIA = EMG_ExpMax_ × burst duration). Muscle burst start and stop times were manually identified from the EMG trace and used to calculate burst duration in % tail beat cycle duration (EMG duty factor) and RIA (a measure of how hard a muscle is working, described above). Timing of body muscle onset relative to the timing of maximum body curvature was calculated as a percentage of the tail beat cycle duration (EMG onset-curvature phase lag). Note that most EMG variables are presented only for the left-side electrodes. EMG data for right-side electrodes are used only to test for differences in timing of contralateral muscle contraction.

### Statistical analysis

Linear statistics and graphing were carried out in R 3.3.1 (R Team Core 2018) using the tidyverse package (Wickham et al. 2019). We calculated trial averages and used these as our observations for all models (each fish performed two to three trials in each condition). Linear mixed-effects models were created in nlme (Pinheiro et al. 2018) with viscosity, position, and the interaction of these two variables (when applicable, based on AIC model comparisons) as fixed effects and individual as a random effect. For those variables that were measured at multiple body positions, the random effects were modeled as body point nested in individual to account for potential differences in variation across body positions. Given the number of individuals in our dataset, we have chosen the most conservative estimation of degrees of freedom for this type of model as defined by Pinheiro and Bates (2000). Results for all linear mixed-effects models are reported as ANOVA-type tables. When necessary, corrections were made for unequal variance of the residuals across body positions using the constant variance function (varIdent) in nlme (Pinheiro et al. 2018). Pseudo-*R*-squared values were calculated for each model using MuMIn (Barton 2018). When necessary, post-hoc multiple comparisons were performed across treatments within a position and across positions within a treatment using the estimated marginal means of each model (Lenth 2019). *P*-values were Bonferroni-corrected based upon the total number of pairwise comparisons performed for each dependent variable. Graphs for linear variables were created using ggplot2 (Wickam 2016). Note that Re values and their estimated marginal means are reported non-transformed for clarity but, to account for non-normal residuals, were log-transformed for statistical analysis.

## Results

### Electrode effects

A paired comparison of swimming in each treatment before and after electrode placement was conducted to quantify the effect of EMG electrodes on kinematic behavior. Following electrode implantation (and accounting for speed as a covariate in the model), pectoral fin range of motion remained constant; however, fin angles at the start of both adduction and abduction were 20° larger and pectoral fin frequency was 0.7 cycle s^–1^ faster. All other kinematic variables collected showed no difference following electrode implantation. Due to the lack of differences across most variables and to pair kinematics with muscle data, we have included only post-EMG surgery trials in this analysis. Individuals were swum across all viscosity treatments to allow paired comparisons.

### Kinematic and muscle activity changes along the body

**Fig. 2:**
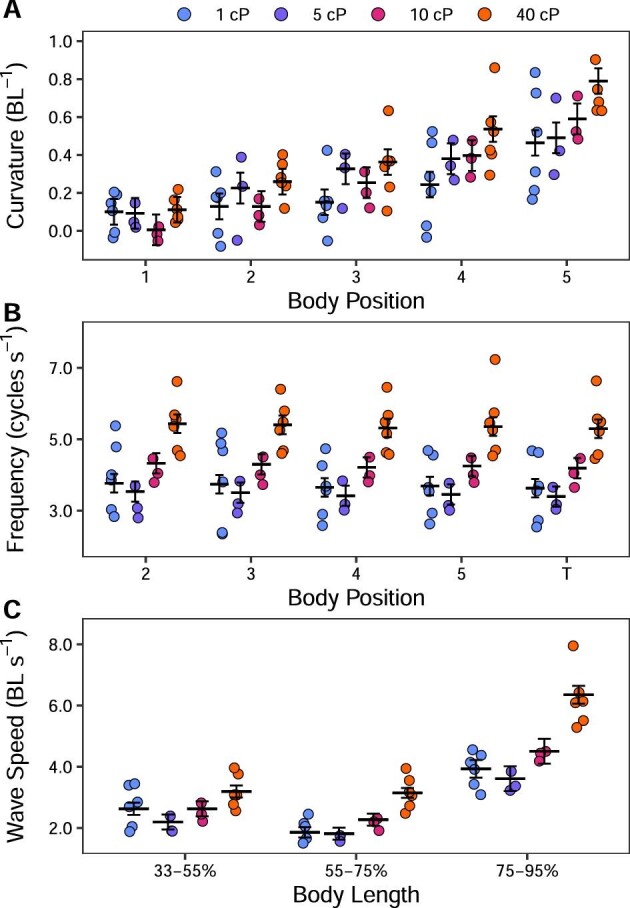
Kinematics of *P. senegalus* swimming in viscous water. Data from the five electrode positions on the left side of the body (as in Fig. 1C) and the tail are shown along the *x*-axis in panels A and B. In panel C, wave speed is shown across three portions of the body of the fish. Curvature, wave speed, and wave frequency increase significantly as viscosity increases at all positions measured (*P* < 0.05).

**Fig. 3:**
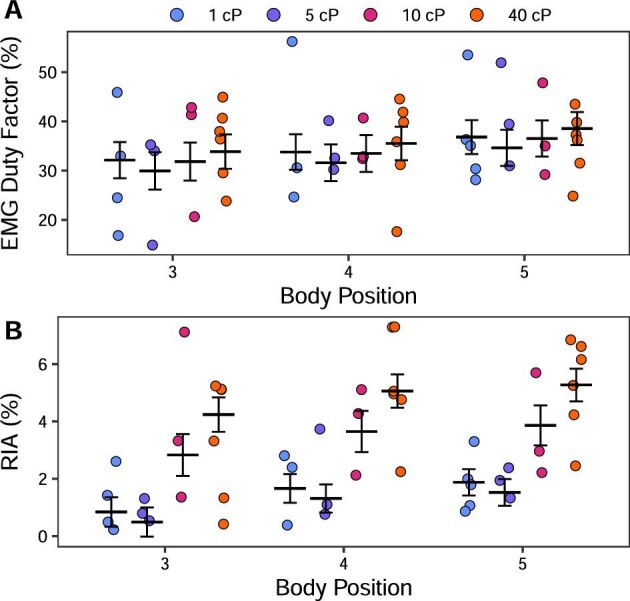
Muscle activity of *P. senegalus* swimming in viscous water. Data from the three most posterior electrode positions (3–5; as in Fig. 1C) on the left side of the body are shown along the *x*-axis. Burst duration (presented in % cycle duration) remains constant as viscosity increases, whereas RIA (presented in % theoretical maximum RIA) increases significantly (*P* < 0.05).

**Fig. 4:**
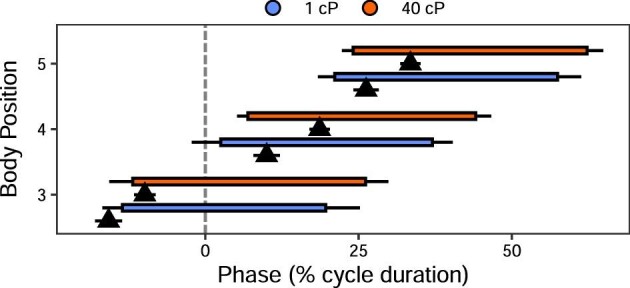
Timing of EMG onset relative to maximum curvature for 1 cP and 40 cP. Mean EMG onset timing is the start of the horizontal bar; mean EMG offset timing is the end of the horizontal bar (standard error is the black line at each end of the muscle activity). Triangle underneath each bar indicates the mean timing of maximum curvature (mean = triangle apex; standard error = horizontal bar on triangle). Start of cycle (0%) is the tail starting to move to the right. Middle of the cycle (50%) is the tail starting to move to the left. Note that phase lag increases toward the posterior of the fish.

In order to understand the anterior–posterior changes in swimming kinematics and motor control, we examined the trends in our variables along the body of the fish. Body curvature increased toward the fish's tail in all treatments (Tables 1 and 2; Fig. 2A). This increase is statistically significant at position 5 in all treatments, and also significant at position 4 in 40 cP. In each treatment, body frequency was consistent across body points (Tables 1 and 2; Fig. 2B). The posterior body (75–95% BL) had a significantly higher wave speed than more anterior body positions (35–55% and 55–75% BL) (except in 5 cP at 35–55% BL; Tables 1 and 2; Fig. 2C). Body EMG duty factor and body muscle RIA did not vary across electrode positions 3–5 in any treatment (Table 1; Fig. 3). EMG onset-curvature phase lag was significantly affected by position according to ANOVA-type results (Table 1), but this difference was not large enough to remain following correction for multiple comparisons (Table 2; Fig. 4).

**Table 1:** Summary of *F* values for linear mixed-effects models fitted to kinematic variables

Variable	*R*^2^*m* [*R*^2^*c*]	Viscosity	Position	Viscosity × position
Speed (BL s^–1^)	0.181 [0.244]	2.74 (3, 29)	n/a	n/a
Re	0.960 [0.962]	305.24* (3, 29)	n/a	n/a
Body curvature (BL^–1^)	0.530 [0.770]	0.84 (3, 143)	9.52* (4,20)	2.57* (12, 143)
Wave speed (BL s^–1^)	0.837 [0.885]	5.87* (3, 81)	30.87* (2, 10)	4.14* (6, 81)
Body frequency (cycles s^–1^)	0.435 [0.634]	70.54* (3, 157)	0.24 (4, 20)	—
Fin frequency (cycles s^–1^)	0.416 [0.605]	12.35* (3, 29)	n/a	n/a
Fin angle—start adduction (rad)	0.161 [0.275]	2.72 (3, 29)	n/a	n/a
Fin angle—start abduction (rad)	0.043 [0.710]	1.47 (3,29)	n/a	n/a
Body EMG duty factor (%)	0.054 [0.516]	0.97 (3, 74)	0.82 (2, 10)	—
Body EMG RIA (%)	0.647 [0.857]	30.34* (3, 74)	1.56 (2, 10)	—
Pectoral fin EMG duty factor (%)	0.011 [0.667]	0.34 (3, 28)	n/a	n/a
Pectoral fin EMG RIA (%)	0.217 [0.357]	3.99* (3, 28)	n/a	n/a
EMG onset-curvature phase lag (%)	0.175 [0.380]	3.17* (3, 73)	7.31* (2,10)	—

*R*^2^*m* is for the fixed effects in the model. *R*^2^*c* is for the whole model. The *F*-value and degrees of freedom (numerator, denominator; in parentheses after each *F*-value) are presented for the fixed effects viscosity, position, and the interaction of viscosity and position. Asterisk denotes *P* < 0.05. Dash indicates a term not included in the model (either due to being used to calculate the dependent variable or based on AIC criterion best fit model). “n/a” is placed in cells where there is only one position possible. Position is the position on the body of the fish and was included in models only for those variables where data were quantified at multiple positions.

**Table 2:** Connecting letter reports based on Bonferroni-corrected *P*-values for kinematic variables across body position, within a given condition

Variable	Viscosity	Position						
		H	1	2	3	4	5	T
Body curvature (BL^–1^)	1 cP	—	a	a	a	ab	b	—
	5 cP	—	a	ab	ab	ab	b	—
	10 cP	—	a	ab	abc	bc	c	—
	40 cP	—	a	a	ab	bc	c	—
EMG onset-curvature phase lag (%)	1 cP	—	—	—	a	a	a	—
	5 cP	—	—	—	a	a	a	—
	10 cP	—	—	—	a	a	a	—
	40 cP	—	—	—	a	a	a	—
		**35–55**	**55–75**	**75–95**				
Wave speed (BL s^–1^)	1 cP	a	a	b				
	5 cP	ab	a	b				
	10 cP	a	a	b				
	40 cP	a	a	b				

Report is given only for the left-side electrode positions; values for right-side electrode positions 2 and 4 are comparable to left-side electrodes 2 and 4. Estimated marginal means and their standard error can be found in Table 3. Position is the position on the body of the fish and was included in models only for those variables where data were quantified at multiple positions.

### Kinematic changes across viscosities

Since the outward kinematics are a combination of motor control and environmental constraint, we report changes in kinematics to understand how successful locomotion in each viscosity was achieved. There was no significant change in swimming speed across all viscosities (Tables 1 and 3), despite a significant decrease in Re (Tables 1 and 3). Fish displayed significantly larger body curvature in 40 cP than in 1 cP for positions 3–5 (Tables 1 and 3; Fig. 2A). There was a significant positive relationship between body frequency and viscosity at all body points at or posterior to position 2 (Tables 1 and 3; Fig. 2B). Wave speed was significantly higher in 40 cP than in 1 cP along the posterior of the fish (Tables 1 and 3; Fig. 2C). Increased viscosity resulted in a higher pectoral fin frequency (Tables 1 and 3). There was no change in fin adduction or abduction angle across viscosities (Tables 1 and 3).

**Table 3:** Connecting letter reports for kinematic and EMG variables across viscosity, within a given condition

		Viscosity			
Variable	Position	1 cP	5 cP	10 cP	40 cP
Speed (BL s^–1^)	—	0.65[0.05]*^a^*	0.42[0.07]*^a^*	0.51[0.07]*^a^*	0.59[0.05]*^a^*
Re	—	11384[1.10]*^a^*	1620[1.09]*^b^*	953[1.10]*^c^*	257[1.10]*^d^*
Body curvature (BL^–1^)	1	0.100[0.068]*^a^*	0.092[0.081]*^a^*	0.005[0.081]*^a^*	0.111[0.067]*^a^*
	2	0.129[0.068]*^a^*	0.225[0.081]*^a^*	0.128[0.081]*^a^*	0.259[0.067]*^a^*
	3	0.151[0.067]*^a^*	0.327[0.081]*^ab^*	0.254[0.081]*^ab^*	0.363[0.067]*^b^*
	4	0.244[0.067]*^a^*	0.380[0.081]*^ab^*	0.397[0.081]*^ab^*	0.537[0.067]*^b^*
	5	0.464[0.067]*^a^*	0.491[0.081]*^a^*	0.590[0.081]*^ab^*	0.790[0.067]*^b^*
Wave speed (BL s^–1^)	35–55%	2.63[0.20]*^ab^*	2.19[0.25]*^a^*	2.63[0.25]*^ab^*	3.19[0.19]*^b^*
	55–75%	1.86[0.17]*^a^*	1.82[0.20]*^a^*	2.27[0.20]*^a^*	3.15[0.16]*^b^*
	75–95%	3.93[0.29]*^a^*	3.61[0.41]*^a^*	4.51[0.41]*^a^*	6.35[0.29]*^b^*
Frequency (cycles s^–1^)	2	3.77[0.26]*^ab^*	3.53[0.28]*^a^*	4.33[0.28]*^b^*	5.43[0.26]*^c^*
	3	3.74[0.26]*^ab^*	3.51[0.28]*^a^*	4.30[0.28]*^b^*	5.40[0.26]*^c^*
	4	3.65[0.26]*^ab^*	3.42[0.28]*^a^*	4.21[0.28]*^b^*	5.32[0.26]*^c^*
	5	3.69[0.26]*^ab^*	3.46[0.28]*^a^*	4.25[0.28]*^b^*	5.36[0.26]*^c^*
	T	3.63[0.26]*^ab^*	3.40[0.28]*^a^*	4.19[0.28]*^b^*	5.30[0.26]*^c^*
	F	5.71[0.26]*^a^*	5.46[0.34]*^a^*	6.11[0.34]*^a^*	7.07[0.26]*^b^*
Fin angle—start adduction (rad)	—	1.48[0.01]	1.48[0.02]	1.45[0.02]	1.44[0.01]
Fin angle—start abduction (rad)	—	1.25[0.04]	1.27[0.04]	1.30[0.04]	1.24[0.04]
EMG duty factor (%)	3	32.1[3.7]	29.9[3.81]	31.8[3.9]	33.9[3.5]
	4	33.8[3.6]	31.6[3.7]	33.5[3.7]	35.5[3.4]
	5	36.8[3.4]	34.6[3.7]	36.5[3.7]	38.5[3.4]
	F	42.1[4.2]	41.1[4.7]	39.1[4.7]	42.5[4.1]
RIA (%)	3	0.84[0.52]*^a^*	0.49[0.51]*^a^*	2.83[0.73]*^b^*	4.24[0.60]*^b^*
	4	1.66[0.50]*^a^*	1.31[0.49]*^a^*	3.65[0.72]*^b^*	5.06[0.58]*^b^*
	5	1.88[0.46]*^a^*	1.52[0.47]*^a^*	3.86[0.70]*^b^*	5.27[0.57]*^b^*
	F	3.52[0.65]*^a^*	3.92[0.87]*^ab^*	4.79[0.87]*^ab^*	5.98[0.63]*^b^*
EMG onset-curvature phase lag (%)	3	1.60[2.49]*^a^*	0.61[2.88]*^a^*	−0.47[2.20]*^a^*	−2.80[2.02]*^a^*
	4	−6.72[2.45]*^a^*	−7.71[2.84]*^a^*	−8.79[2.13]*^a^*	−11.12[1.96]*^a^*
	5	−4.70[2.40]*^a^*	−5.69[2.84]*^a^*	−6.77[2.12]*^a^*	−9.10[1.95]*^a^*

Report is given only for the left-side electrode positions; values for right-side electrodes at positions 2 and 4 are comparable to left-side electrodes 2 and 4. H, head; T, tail; F, right pectoral fin. Estimated marginal means are presented with their standard error (calculated based on the number of trials in each viscosity) in square brackets. Connecting letters are in superscript following the standard error. Position is the position on the body of the fish and was included in models only for those variables where data were quantified at multiple positions.

### Electromyography changes across viscosities

EMG magnitude and timing were quantified to determine whether the motor pattern changed due to increased viscosity. Quantitative data are only presented for those electrode positions where there was enough data in all viscosities for complete linear models, as described above. Since burst presence at positions 1 and 2 during swimming in 1 cP and 5 cP is inconsistent (present in <10% of the tail beat cycles analyzed in each condition), EMG data were analyzed for only positions 3 through 5 (Fig. 1). Body EMG duty factor did not change across viscosities (Tables 1 and 3; Fig. 3A). Body RIA increased significantly in 10 and 40 cP for all applicable body electrode positions, while pectoral fin RIA increased significantly in 40 cP (Tables 1 and 3; Fig. 3B). EMG onset-curvature phase lag was significantly affected by viscosity in the ANOVA-type results (Table 1), but all differences became nonsignificant after correction for multiple comparisons (Table 3; Fig. 4). Note that in support of a unilateral, alternate pattern of body muscle activity that typifies swimming (in other words no presence of co-contraction in a more viscous system), neither left- nor right-side electrodes show any indication of a change in body EMG duty factor (consistently <50% cycle duration; linear mixed-effects model, *P* > 0.05) or EMG onset timing (as a percentage of tail beat cycle duration; linear mixed-effects model, *P* > 0.05) across viscosities.

## Discussion

Computer simulations of swimming that maintain motor control patterns but increase water viscosity show that swimming speed, body amplitude, and body frequency decrease in the absence of sensory feedback (Tytell et al. 2010). In short, an increase in viscosity dampens the system. If living systems were without sensory feedback, we would expect to see the same dampening of swimming kinematics. In contrast, *P. senegalus* maintain their swimming speed even when water is 40× more viscous than normal and do so by increasing their body and pectoral fin muscle effort (RIA). Our results are in line with the few previous studies that have shown adult fish maintain swimming speed in viscous water (Johnson et al. 1998; Horner and Jayne 2008; Danos and Lauder 2012), and accord with clear evidence that some fishes have a preferred swimming speed related to energy consumption (Beamish 1978). The changes in muscle activity we report for *P. senegalus* are similar to those in lungfish (the only other investigation of fish muscle activity in viscous water), suggesting that these two phylogenetically distinct animals respond to this novel environment in similar ways. The maintenance of swimming speed in living fish suggests sensory feedback systems fine-tune motor control to address the constraints of this novel environment.

### Vision

Fish use vision to facilitate steady swimming in both flowing and still water (reviewed in Liao 2007). Visual flow allows fish to perceive swim speed relative to their surroundings and thus helps tune kinematics and muscle activity to achieve a preferred swimming speed (Ahrens et al. 2012). Indeed, migrating salmonids appear to maintain their ground speed at roughly 0.5 BL s^–1^ regardless of the river flow velocity they encounter (Beauchamp et al. 1989). Increasing viscosity does not remove visual cues and *P. senegalus* maintained voluntary swimming speed despite the increase in mechanical constraint, suggesting that despite poor visual acuity (Znotinas and Standen 2019), visual feedback could be used by *P. senegalus* to determine preferred swim speed.

### Lateral line

How other sensory feedback systems modify swimming performance in high viscosity requires closer examination of the body kinematics and muscle timing. In high viscosity, *P. senegalus* increase their body curvature, the frequency of their pectoral fin and body motions, and the speed of the body wave as it travels posteriorly. Each of these kinematic variables has the capacity to impact the environmental signals received by mechanical sensory feedback systems. For example, lateral line cells detect flow velocity and acceleration along the fish's length that can be used to tune swimming movements (Engelmann et al. 2000; Coombs et al. 2001; McHenry et al. 2008; Bleckmann and Zelick 2009). The velocity and thickness of the boundary layer around the fish directly affect the signal received by superficial neuromasts (i.e., they are speed sensitive), and indirectly affect canal neuromasts (i.e., they are acceleration sensitive) (van Netten 2006; McHenry et al. 2008; Rapo et al. 2009; Windsor and McHenry 2009). In a more viscous (lower Re) environment, the boundary layer is expected to be larger and thus more similar to that of a slowly swimming fish (Windsor and McHenry 2009). An increase in body motion (both increased curvature and wave speed) in high viscosity would cause an increase in local flow speed along the body, and should decrease the boundary layer surrounding the fish, increasing feedback to the lateral line cells. High-frequency body undulations may have the same effect, increasing the response of lateral line cells (van Netten 2006; McHenry et al. 2008). While there are mixed reports about the role of efferent neurons working to filter self-generated hydrodynamic signals during steady swimming (e.g., evidence for such activity [Roberts and Russell 1972] and against [Mensinger et al. 2019]), it does appear that the lateral line of fish is responsive to normal swimming body movements. These signals may either be interpreted as a source of information (e.g., when body movements are unexpected) or as a source of noise (e.g., when body movements are expected), depending on the situation (Montgomery et al. 2009). Both afferent and efferent lateral line signaling evidently require further investigation, but they present potentially important sources of information for fish swimming in an increased viscosity environment. The role of lateral line feedback in the observed responses could be tested by swimming *P. senegalus* in viscous water following a block of their lateral line cells. If lateral line sensory feedback is important, as we propose here, we would expect fish with a blocked lateral line in a high viscosity environment to have no change in muscle activity, and therefore demonstrate a dampened kinematic output.

### Mechanical constraint

Independent of sensory feedback, mechanical constraint most likely contributes to kinematic differences between water and high viscosity and is best represented by the phase lag between muscle activation (i.e., EMG onset) and resultant body motion. Based on a model of undulatory swimming, an increase in this phase lag can be a direct consequence of interactions between relatively high fluid forces and relatively weak body forces (e.g., muscle force, spring forces), as is the case in high viscosity environments (Tytell et al. 2010). Presumably, the increased phase lag results because (for a given muscle force) the time interval between initiating muscle activity and resultant kinematic output should be inversely related with the resistive forces caused by the viscosity of the environment. Thus, the environment constrains the timing of kinematic output. Although not statistically significant (Table 1; Fig. 4), this constraining effect is present, as despite increases in muscle effort (which might balance environmental resistance), EMG onset-curvature phase lag tends to increase in *P. senegalus* as viscosity increases. A significant increase in phase lag is achieved in lungfish when viscosity reaches 100 times that of water (Horner and Jayne 2008). Since our highest viscosity is only 40 times more viscous than normal water, it is possible that we would see a statistically significant magnitude shift in *P. senegalus* at higher viscosities. Interestingly, our fish were swimming at similar Re in 40 cP water to lungfish moving in ∼100 cP water. Thus, the dynamics of these systems are similar, suggesting that something other than simply mechanical constraint is influencing the observed phase lag.

Whether the change in EMG onset-curvature phase lag observed in both lungfish and *P. senegalus* is solely due to passive mechanical constraint, is unclear from intact animal data; the observed lag could be contributed to active shifts in body stiffness as well. Regardless, the functional consequences of this shift remain the same. Shifting EMG onset earlier in the curvature cycle (assuming consistent EMG duty factor as in our dataset) changes the amount of positive work done by the muscles. While body curvature is not a perfect proxy for muscle length change timing, shifting EMG onset earlier relative to maximum curvature makes it more likely that muscles are active during lengthening. If present, negative work would help stiffen the body of the fish and facilitate power transfer to the posterior of the body while swimming through a high resistance fluid. The larger lag in lungfish than in *P. senegalus* in similar Re environments may be due to the dermis and scales of *P. senegalus* stiffening the fish more than the skin of lungfish (Long et al. 1996). If so, *P. senegalus* would have less need to actively stiffen the body to permit effective transfer of power to the caudal fin for swimming and so would show a smaller increase in phase lag between EMG onset and max body curvature.

### Similarities between high viscosity and high-speed swimming

A lag between EMG onset and max curvature is also observed during high-speed swimming in a variety of fishes (e.g., Jayne and Lauder 1995a; Coughlin 2000), suggesting high viscosity swimming is coopting a natural swimming control pattern. Generally, when fish swim faster, wave speed, tail beat frequency, pectoral fin beat frequency (when present), and muscle activity increase (to facilitate the more powerful movements) while maintaining a unilateral, alternate pattern of muscle activity along the body (e.g., Jayne and Lauder 1995a, 1995b; Coughlin 2000). As speed increases, absolute body EMG burst duration decreases to maintain duty factor with an increased tailbeat frequency (Shadwick et al. 1998). Interestingly, as water viscosity increased, *P. senegalus* showed these same changes in kinematics and muscle activity but without an accompanying change in speed (Tables 1 and 3; Figs. 1–3). Note that because the observed increase in wave speed occurs without a concurrent increase in swimming speed (i.e., slip or propeller efficiency decreases), it suggests that swimming in viscous water is less efficient than swimming in normal water.

Elongate fish including eels, needlefish, and gar (Long et al. 1996; Liao 2002; Tytell 2004) have shown a positive relationship between swim speed and body amplitude. Since *P. senegalus* are also elongate and increase body curvature (and amplitude) at increased viscosities, this may also suggest *P. senegalus* coopt changes in motor control typically used to increase swim speed. However, whether a positive relationship between body amplitude and swim speed is observed in *P. senegalus* remains to be determined. *Polypterussenegalus* also increase the frequency of pectoral fin cycles in high viscosity, but keep angle at the start of adduction and abduction constant, a change typical of Labriform propulsion as speed increases (e.g., Drucker and Jensen 1996; Walker and Westneat 1997). *Polypterus senegalus* may move the pectoral fin in the same arc because at these muscle lengths, despite changes in fin cycle frequency, both work and power output are maximal (e.g., seahorse dorsal fin muscle; Ashley-Ross 2002). Deviating from this range of motion would therefore diminish the ability to overcome the increased resistance of a high viscosity environment or to increase swimming speed. Thus, the changes in kinematics and muscle activity of *P. senegalus* while swimming in viscous water mirror changes observed when fish increase swimming speed.

### Possible local sensory feedback mechanisms

Increasing viscosity increases the mechanical dampening of the environment acting on sensory systems. Most likely, sensory information from local (proprioception) and higher order (vision and lateral line) systems work in concert to achieve the exaggeration of body and fin swimming motions we see in *P. senegalus* experiencing higher viscosity. While there is still little direct evidence for proprioceptive senses in bony fish (but see Henderson et al. 2019; Aiello et al. 2020), several studies have demonstrated stretch-receptive cells in the spinal cord of lamprey and dogfish (Grillner et al. 1981a, 1981b, 1984; Grillner and Wallén 1982). These cells change firing frequency in response to local bending angle and velocity (Massarelli et al. 2017). Both stretch receptors and proprioceptors input directly into local circuits (and stretch receptors also provide feedback directly to the reticulospinal neurons), facilitating rapid adjustment of locomotor movements to changes in environment (Hsu et al. 2016). In an environment with increased mechanical resistance, like that caused by an increase in viscosity, a given intensity of muscle activity would result in less local bending (Tytell et al. 2010). The observed increased muscle activity in *P. senegalus* swimming in high viscosity water may have been the result of feedback from stretch receptors or proprioceptors signaling the need for increased local bending. The fact that increased muscle activity resulted in an increase and not just the maintenance of local bending suggests that if such local sensory feedback exists in *P. senegalus* it was either overcompensating or working in combination with other sensory feedback systems to augment movement. Indeed, whether stretch-receptive or bending-sensitive proprioceptive cells are present in *P. senegalus* remains to be seen and confirmation would require direct cell recordings at different levels of bending, as has been done in dogfish and lamprey (Grillner et al., 1981a, 1981b, 1984; Grillner and Wallén 1982).

### Conclusion

Mathematical models suggest that without sensory feedback, increased mechanical constraint at high viscosity will result in dampened swimming kinematics. The results we present here suggest that in living animals, sensory-driven changes actually increase swimming kinematics in a high viscosity environment. These results suggest that manipulating viscosity in combination with alteration to different sensory feedback systems could shed light on key sensory inputs that impact motor control in novel environments.

## Supplementary Material

obab024_Supplemental_Files

## Data Availability

Dataset and R code used for statistical analysis are included as supplementary files. Additional data are available upon request.
